# A Scoping Review of Machine Learning-Based Prediction of Alzheimer’s Disease Using Blood Biomarkers

**DOI:** 10.1177/11795972261481838

**Published:** 2026-08-26

**Authors:** Muhammad Hamza Rafique Bhatti, Amir Aly, Asiya Khan, Shakil Awan

**Affiliations:** 1 School of Engineering, Computing and Mathematics, 6633 University of Plymouth, Plymouth, UK

**Keywords:** blood biomarkers, machine learning, Alzheimer’s disease, healthcare applications, disease prediction, early diagnosis, graphene, field effect transistor sensors

## Abstract

**Background:**

Alzheimer’s disease (AD) is a progressive neurodegenerative disorder of late life that causes cognitive and functional decline and substantial mortality. Machine learning (ML) is increasingly used to discover patterns in clinical and biomarker data that support earlier and more accurate AD detection.

**Objectives:**

This scoping review addresses three core research questions. First, we investigate recent trends in using machine-learning techniques to detect Alzheimer’s disease using blood biomarkers. Second, we identify the blood biomarkers involved in Alzheimer’s detection and evaluate how machine learning has been applied to improve the diagnostic capabilities of these biomarkers. Third, we highlight significant challenges associated with using machine learning for blood biomarker data in Alzheimer’s detection and examine proposed advancements or solutions to handle these problems.

**Methods:**

In June 2025, we searched six academic databases to identify relevant papers on blood biomarkers and ML methods for Alzheimer’s Disease. Search queries were developed based on our predefined research questions. Papers were then screened using defined inclusion and exclusion criteria, where titles, abstracts, and full texts of articles were systematically reviewed.

**Results:**

Following the screening approach, we selected 36 papers that fulfilled our inclusion and exclusion criteria. Through careful examination, we classified blood biomarkers into four types: transcriptomics, proteomics, multi-omic biomarkers, and general elemental blood biomarkers. Across these studies, proteomic blood biomarkers consistently emerged as significant indicators for Alzheimer’s disease, including Alpha-2-Macroglobulin (A2M), Apolipoprotein E (ApoE), Eotaxin-3 (EOT3), plasma phosphorylated tau (p-tau 181), and neurofilament light chain (NfL). Furthermore, we explored challenges such as small sample sizes, lack of standardization, heterogeneity, and data imbalance.

**Conclusions:**

This review provides insights into how combining blood biomarkers with ML can enhance AD prediction. The review summarizes key challenges and identifies critical gaps for future research.

## 1. Introduction

### 1.1. Background

Alzheimer’s Disease (AD) is one of the most significant neurodegenerative disorders and is currently the sixth-leading cause of death worldwide. It affects millions of people globally, posing significant challenges for healthcare systems. The complex nature of brain diseases necessitates comprehensive evaluation and extended timeframes for accurate diagnosis and prediction.^1,2^ Dementia impacts roughly 5–8% of people over the age of 60, according to the World Health Organization (WHO). As of September 2020, about 50 million people worldwide were living with dementia, with an estimated 10 million new cases arising each year (World Health Organization, 2020).^3^ AD, the most dominant condition of dementia, is characterized by its irreversible nature. This chronic neurodegenerative disorder is caused by the irregular accumulation of proteins in the brain, including amyloid-beta plaques and tau tangles, which disrupt neuronal communication and severely affect the patient’s mental and physical health. The pathological changes lead to brain atrophy, reducing brain volume and degenerating neural links. As the brain shrinks, neuronal damage and loss of interconnectivity also worsen the condition.^4^ Cognitive decline, particularly memory loss, impairs short-term memory and slows vital cognitive functions such as language processing and reasoning. Therefore, patients usually become increasingly dependent on caretakers for day-to-day activities.^5^

Since there is currently no absolute treatment for AD, healthcare professionals highlight preventive measures to slow disease progression. Diagnosing AD traditionally involves comprehensive behavioral and cognitive testing, as well as a thorough assessment of the patient’s medical record. These assessments are typically performed over an extended duration. The diagnosis of AD has been based on the concentration of tau and amyloid proteins.^6^

Advanced brain imaging procedures, such as magnetic resonance imaging (MRI) and positron emission tomography (PET), are used to assess structural or pathological changes in the brain. Also, cerebrospinal fluid (CSF) testing through lumbar puncture is utilized to measure amyloid and tau levels. Regardless, these approaches have several limitations. Amyloid-based biomarkers mainly focus on amyloid concentrations and provide limited information about tau pathology and neuroinflammation.^7^ Similarly, these tests are usually unable to detect AD in its earliest phases because amyloid protein concentrations are minimal at that time. By the time amyloid levels immensely increase, the disease has often progressed to an irreversible phase, yielding comprehensive neuronal damage. In primary care settings, these diagnostic methods are also associated with high costs, limited accessibility, and invasiveness. MRI and PET require specialized technical equipment that is not widely available, and the lumbar puncture procedure can increase patient stress and carry a risk of infection.^8^

Due to the high costs and invasive nature of amyloid-based diagnostic methods, researchers have shifted focus toward developing less costly and more accessible diagnostic techniques for AD. Blood biomarkers have emerged as an alternative, proposing non-invasive, cost-effective, and widely available diagnostic solutions.^9^ Blood tests are already a common healthcare approach, causing their integration into AD diagnosis to be both applicable and feasible. These biomarkers deliver valuable insights that, when integrated with cognitive and behavioral assessments, particularly improve diagnostic accuracy. The use of blood biomarkers decreases healthcare costs and eases the financial burden on patients.^10^

Blood biomarkers contribute substantially to the efficiency of the healthcare system. Moreover, they enable early detection of AD by identifying metabolic changes that occur before the pathological accumulation of amyloid and tau proteins in the brain.^11^ Studies have identified several blood biomarkers that indicate molecular and biological modifications in AD patients. For example, MicroRNA (miRNA) and messenger RNA (mRNA) extracted from blood provide precise insights into disease progression. Proteins in blood samples are studied to examine biological alterations, while blood gene expression helps trace genomic changes.

Transcriptomic blood biomarkers involve examining RNA molecules to assess gene expression levels in the blood. Changes in gene expression profiles have been shown to reflect AD progression. miRNA and mRNA from blood samples are evaluated to determine the concentrations and patterns of gene expression associated with disease development. Similarly, proteomic blood biomarkers capture alterations in protein expression levels. Specific changes in protein concentrations in the blood have been linked to AD progression. Proteomic profiling enables the investigation of protein variations across different stages of the disease, as protein levels fluctuate with AD progression. Omic studies (proteomics, metabolomics, transcriptomics) utilize high-throughput platforms to profile thousands of biological molecules to discover novel biomarkers and dysregulated pathways. In contrast, protein biomarkers are also detected by immunoassays (ELISA, multiplex bead arrays, and electrochemiluminescence platforms) that use antibody-based methods to measure specific and pre-defined proteins. In multi-omics blood biomarkers, multiple biomarkers are utilized simultaneously to investigate their interrelationships and potentially improve AD treatment. This integrative approach provides a more comprehensive understanding of AD and may support the development of more effective treatment strategies. Additionally, elemental and general clinical blood biomarkers are widely used in clinical practice and are considered potential indicators of AD progression. Elemental biomarkers comprise trace elements, such as minerals and metals. However, general clinical biomarkers show physiological and metabolic changes in the blood. The differences in elemental concentrations may contribute to the onset and progression of neurodegenerative diseases, including AD.

Additionally, blood serum and plasma are utilized to identify additional biochemical mutations related to AD. Qin et al^12^ investigated 146 plasma proteins and recognized 13 biomarkers that are effective for the early prediction of AD. Similarly, Abdullah et al^13^ used a blood-based transcription dataset. They found biomarkers such as CST7, DPAGT1, DZIP1, MOSC1, TACC3, and YDJC to be vital for AD diagnosis. Additionally, a study by Ref. 14 investigated the use of blood serum and plasma to enhance diagnostic accuracy, identifying four serum biomarkers (Serum_IL6, Serum_IL7, Serum_sTNFR1, and Serum_sVCAM1) and six plasma biomarkers (Plasma_TPO, Plasma_Eotaxin3, Plasma_SAA, Plasma_IL6, Plasma_sTNFR1, and Plasma_sICAM1) as the effective for predicting AD.

The healthcare sector yields extended volumes of patient data, including raw data, images, and diagnostic reports. AD patients experience multiple diagnostic methods, contributing to the collection of such data. ML plays a key role in interpreting these data and forecasting the progression of AD.^8^ By recognizing hidden patterns within datasets, ML shows associations between features, effectively mapping between dependent and independent variables.^15^ ML models allow the early prediction of AD, which is vital for delaying disease progression. When trained on blood biomarker datasets, ML models can accurately identify AD, assisting in accurate and convenient diagnosis.^16^ This integration of ML with blood biomarkers not only improves diagnostic accuracy but also lessens the time and expenditure associated with traditional diagnostic methods. Similarly, ML-based models assist medical practitioners by enhancing diagnostic accuracy and efficiency, thereby reducing the burden on healthcare systems.

In a study by,^12^ ML algorithms, including Random Forest (RF), Logistic Regression, Naïve Bayes, Support Vector Machine (SVM),^17^ and CatBoost, were applied to plasma protein for early AD detection. Among these, CatBoost achieved the highest performance, with an AUC of 0.900. Similarly, Yuan et al^3^ employed ML models such as SVM, AdaBoost, RF, and neural networks to identify optimal blood biomarker panels and design predictive models for accurate AD diagnosis. Furthermore, recent advances in graphene-based biosensing have demonstrated the feasibility of detecting the AD biomarker Clusterin using graphene field-effect transistors (GFETs), achieving limits of detection as low as approximately 300 fg/mL (4 fM).^18^ These findings emphasize the significance of ML algorithms in enhancing diagnostic procedures when paired with blood biomarkers. By leveraging ML, healthcare practitioners can decrease diagnostic time and effort while achieving high levels of accuracy.

### 1.2. Rationale

Previous studies, such as,^19^ emphasize fluid biomarkers—including blood, CSF, and saliva and often integrate them with neuroimaging approaches. However, prior reviews predominantly focus on CSF and saliva, with comparatively fewer examining blood biomarkers. While some studies address invasive diagnostic approaches, they rarely evaluate the effectiveness of blood biomarkers specifically for AD. Moreover, when blood biomarkers are discussed, they are frequently framed in relation to neuroimaging, overlooking their broader standalone diagnostic potential. To address this gap, this scoping review systematically maps machine learning applications to blood biomarkers for early AD detection.

### 1.3. Objective

The primary objective of this research is to summarize the application of ML techniques with blood biomarkers for AD diagnosis. Mainly, this research aims to address the following research questions:

(i) What are the key recent research trends in using machine learning for Alzheimer’s disease detection with blood biomarkers?
(ii) Which blood biomarkers have been explored in studies on Alzheimer’s detection, and how have machine learning methods been applied to analyze and enhance their diagnostic utility?
(iii) What are the significant challenges in applying machine learning to blood biomarker data for Alzheimer’s detection, and what improvements or solutions have been proposed?

## 2. Methods

### 2.1. Protocol

This research adhered to the PRISMA-ScR (Preferred Reporting Items for Systematic Reviews and Meta-Analyses Extension for Scoping Reviews)^20^ guidelines and utilized the PICO framework. These methods were adopted to provide a clear understanding of the scoping review while effectively showing the broader concepts of blood biomarkers and machine learning in Alzheimer’s disease prediction. The primary purpose was to analyze recent studies that emphasize the integration of blood biomarkers with machine learning methods to enhance the diagnosis of Alzheimer’s disease.

### 2.2. Eligibility Criteria

The research questions were prepared using the Population, Intervention, Comparison, and Outcome (PICO) framework. This approach enables alignment with the study objectives and helps the formulation of inclusion and exclusion criteria. In the Population section, the emphasis is on patients with Alzheimer’s disease. The Intervention section includes machine learning approaches applied to blood biomarkers. Accordingly, the review focuses on recent advances in the application of machine learning techniques to blood biomarker data. In the Outcome section, the focus is on identifying emerging research dedicated to the integration of blood biomarkers and machine learning models, with a thorough investigation of all blood biomarkers in the diagnosis of Alzheimer’s disease. The PICO framework is shown in Table 1.

**Table 1.** PICO (Population, Intervention, Comparison, and Outcome) Criteria and Definitions

PICO criteria	Definition
Population	Patients with Alzheimer’s disease (AD)
Intervention	Application of machine learning models on blood biomarkers
Comparison	No specific comparison is required
Outcomes	Identification of emerging research trends on ML integration with blood biomarkers for AD diagnosis

### 2.3. Search Strategy

The research articles published between 2020 and 2025 were selected from research databases. Six major databases were selected for this purpose: IEEE Xplore, PubMed, Scopus, ScienceDirect, Web of Science, and ACM Digital Library. After conducting the primary searches in selected databases, we used Google Scholar to hand-search for additional relevant studies, including checking reference lists of included articles. Google Scholar was not considered a core database due to its limited reproducibility. These databases were specially selected based on their applicability to the healthcare and computer science domains. Google Scholar provides a broad scope of search results, including scientific reports, conference papers, and research studies. IEEE Xplore focuses on the application of artificial intelligence (AI) models in the healthcare sector. PubMed emphasizes biomedical applications and clinical research. Scopus and Web of Science present a comprehensive scope of ML applications in the biomedical literature. ACM Digital Library specializes in computing and information technology, while ScienceDirect encompasses a combination of research articles in medical and technical journals, especially those investigating the intersection of AI and healthcare. By incorporating these various databases, we were able to identify studies that discuss the use of AI and blood biomarkers for diagnosing AD. After selecting the research databases, a search query was created to retrieve relevant papers. The query was then adapted to the specific syntax and fields of each database. The searches were conducted in June 2025 across all databases. The query was carefully formed to focus on blood biomarkers and ML for AD prediction. It also contained documents that used ML or AI techniques for the prediction of AD. By incorporating the term “Alzheimer’s disease”, the query ensured that the outcomes were limited to studies focused only on AD, excluding unrelated diseases or conditions. The list of research keywords used in the query is summarized in Table 10.

### 2.4. Inclusion Criteria

Studies were assessed using predefined inclusion criteria to ensure relevance to the research objectives. The criteria specifically targeted studies focusing on blood biomarkers and the application of machine learning for Alzheimer’s disease prediction. Studies were included if they met the following inclusion criteria:

1) Studies focusing on the detection or diagnosis of Alzheimer’s disease using blood biomarkers.
2) Studies applying machine learning techniques to analyze the blood biomarker data.

### 2.5. Exclusion Criteria

Studies were excluded if they met the following conditions:

1) Did not focus on blood biomarkers for Alzheimer’s disease detection or diagnosis.
2) Did not apply machine learning techniques to blood biomarker data.
3) Were not peer-reviewed (e.g., preprints, white papers, or technical reports).
4) Lacked empirical evidence (e.g., position papers, purely theoretical discussions without data).
5) Did not report clear evaluation metrics for model performance.
6) Were not published in English.

### 2.6. Screening and Article Selection

Articles were retrieved from multiple publication databases, covering various types of sources, including conference proceedings, journal articles, and review papers. In the first screening stage, titles were reviewed. Subsequently, abstracts were screened to assess study relevance and eligibility based on the inclusion criteria. Finally, full-text articles were thoroughly reviewed to confirm eligibility and extract key information. This multi-stage screening process resulted in the final selection of studies included in the scoping review. The abbreviation and their definitions are shown in Table 11.

### 2.7. Data Extraction

After the final selection of 36 papers, key details were systematically extracted and organized into a spreadsheet. For each paper, the recorded information included publication details (title, authors, journal, and year), the type of blood biomarker studied, ML algorithms applied, datasets used, performance metrics, study objectives, and methodologies. All sections of the papers were critically examined to ensure comprehensive data extraction. This review also categorized the types of blood biomarkers investigated, contributing to a deeper understanding of their roles in Alzheimer’s disease detection. In all 36 papers, ML models were emphasized and applied to analyze blood biomarkers. Most studies used supervised learning, an ML technique, to predict AD. In supervised learning, models are trained on labeled data and evaluated on unseen data. The input variables represent patient features, while the output variables correspond to diagnostic outcomes. The model learns patterns linking the input features to the corresponding outputs. Once trained, the model’s predictive accuracy is evaluated using unseen testing data.

### 2.8. Data Analysis and Synthesis

The selected papers and the extracted spreadsheet data served as the primary sources for analysis. Through a critical investigation of the selected papers, a synthesis was developed by extracting relevant information from each study to highlight relationships among various types of blood biomarkers associated with Alzheimer’s disease. A comparative analysis was conducted to examine recent trends in the use of blood biomarkers and machine learning algorithms for Alzheimer’s disease prediction. Similarly, challenges and limitations in applying machine learning algorithms to blood biomarker data were thoroughly investigated. The study also identified research gaps, providing valuable insights for future research.

## 3. Results

### 3.1. Overview of Included Studies

**Figure 1.**
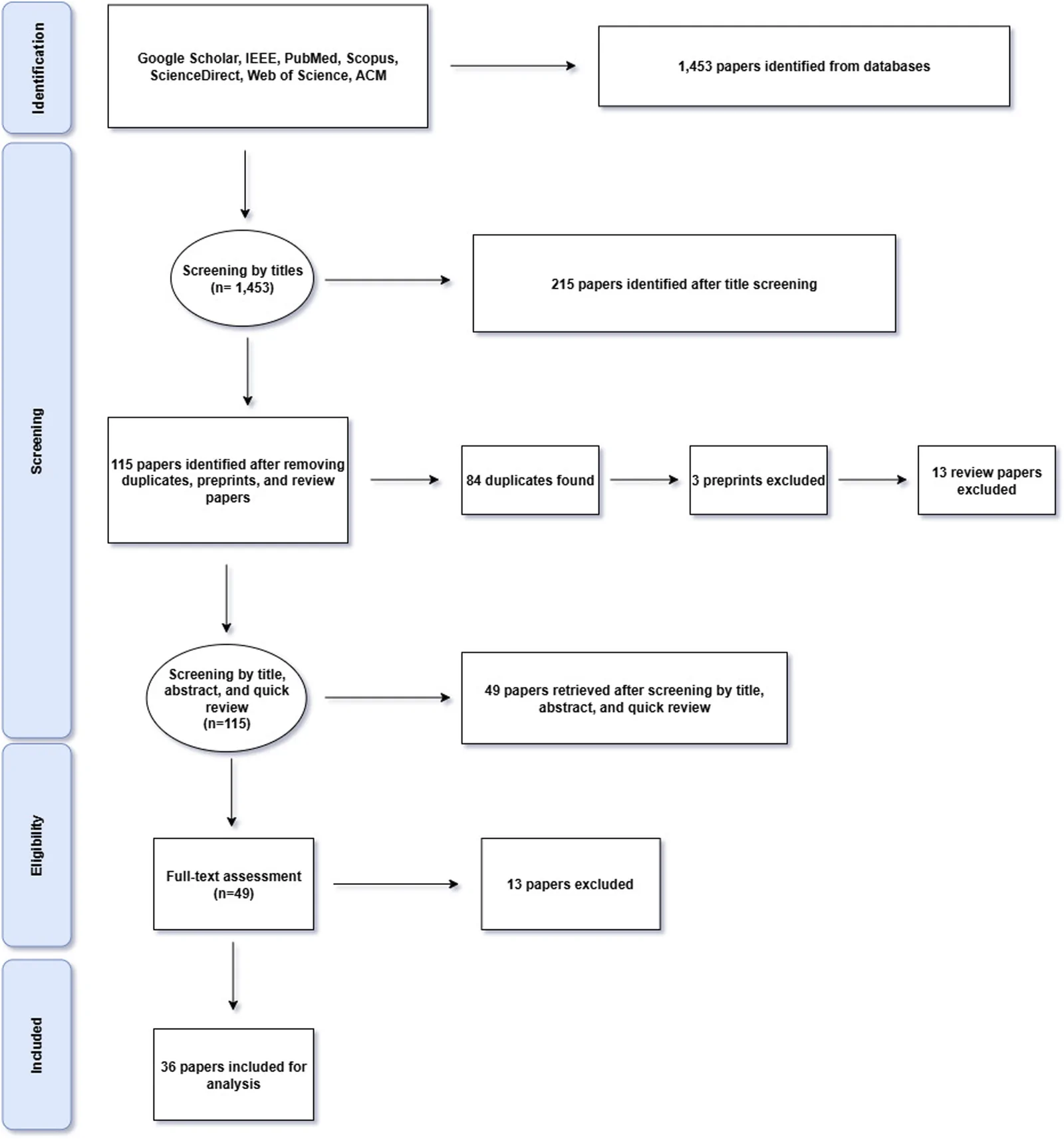
PRISMA flow diagram of the scoping review

The search across databases yielded a total of 1,453 records. Google Scholar retrieved 1,000 papers, while IEEE yielded six papers. PubMed provided 51 papers, and Scopus and ScienceDirect returned 63 and 14 papers, respectively. Web of Science yielded 53 papers, and ACM provided 10 papers. However, these studies consist of journal articles, conference proceedings, review papers, scientific reports, and non-peer-reviewed papers. In the first phase of screening, titles and keywords were scanned to identify potentially suitable studies. After the initial title-based screening, 215 papers out of the 1,453 were selected for further evaluation. The title-based screening indicated that IEEE, PubMed, Scopus, and Web of Science contributed the majority of papers aligning with the specified inclusion and exclusion criteria. In the second screening stage, 84 duplicate papers were removed to eliminate redundancy from the initial set of 215 papers. After removing the duplicates, 131 papers remained. Further screening led to the exclusion of three preprint papers and 13 review articles. This screening stage resulted in 114 papers selected for further evaluation. In the third stage, papers were assessed based on their titles, abstracts, and overall content review. After detailed screening, 49 papers were included from the 114 records. In the final stage, a comprehensive full-text evaluation was conducted. This stage enabled the final confirmation and refinement of the selected studies. After this extensive review, 13 papers were excluded, resulting in a final set of 36 papers included in the scoping review. The complete detailed procedure is illustrated in Figure 1.

### 3.2. Characteristics of Participants

In the 36 selected papers, most patients were over the age of 60. The participants were commonly classified into three groups: Healthy Controls (HC), patients with Alzheimer’s disease (AD), and individuals with Mild Cognitive Impairment (MCI). Medical records were collected, and multiple diagnostic tests were performed on the participants. The selected studies primarily focused on classification tasks aimed at improving diagnostic accuracy and precision using blood biomarkers and machine learning models. Furthermore, the studies analyzed blood biomarkers to identify the most predictive features for Alzheimer’s disease diagnosis. Attention was devoted to feature selection and extraction techniques, with several studies emphasizing the use of explainable AI methods to interpret model outcomes. These 36 papers represent valuable insights into the integration of blood biomarkers with machine learning for Alzheimer’s disease diagnosis.

## 4. Discussion

### 4.1. Principal Findings

**Figure 2.**
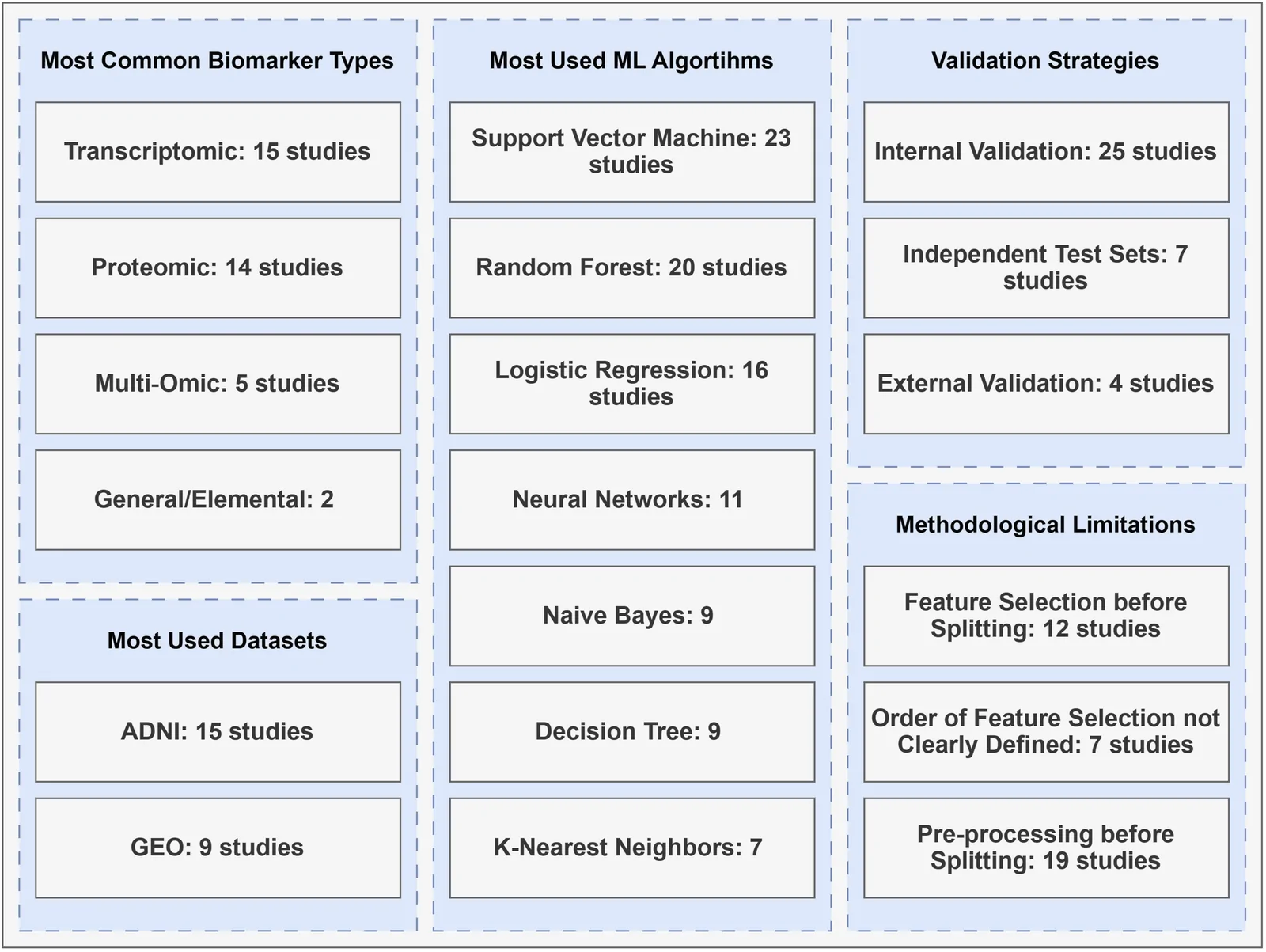
Overview of key findings

This scoping review investigates the application of ML algorithms with blood biomarkers for AD prediction. The authors systematically searched research databases to identify studies involving blood biomarkers and ML. The review encompasses various types of blood biomarkers, including proteomic, transcriptomic, multi-omic, elemental and general clinical blood biomarkers. Non-invasive approaches, such as those using blood biomarkers, offer cost-effective alternatives for disease diagnosis. The application of ML algorithms has proven highly valuable, particularly in the healthcare sector. This review examines various ML algorithms that assist healthcare practitioners in enhancing diagnostic accuracy and supporting clinical decision-making. An overview of the key findings of this review is illustrated in Figure 2.

### 4.2. Blood Biomarkers

This study reviewed various types of blood biomarkers used in AD prediction. Of the 36 studies analyzed, 15 focused on transcriptomic biomarkers, while 14 investigated proteomic biomarkers for AD detection. Two studies explored general clinical blood biomarkers and elemental biomarkers. Additionally, five studies adopted multi-omic approaches, integrating multiple types of blood biomarkers to improve diagnostic performance.

#### 4.2.1. Transcriptomic Blood Biomarkers for AD Detection

Transcriptomic biomarkers consist of RNA molecules that provide insights into gene expression within the blood. mRNA and miRNA reflect temporal changes in gene activity. Dysregulated gene expression linked to neuroinflammation, immune activation, and oxidative stress can serve as a potential indicator for AD diagnosis. A detailed summary of transcriptomic blood biomarkers is summarized in Table 2. A total of 15 studies investigated RNA-based biomarkers and used ML algorithms that analyze RNA expression patterns in transcriptomic blood biomarkers. Optimal gene features are extracted from large RNA expression profiles to uncover complex relationships within AD. These studies demonstrate how RNA biomarkers enhance predictive capabilities for Alzheimer’s disease diagnosis. A summary of ML model applications to transcriptomic biomarkers is summarized in Table 3.

**Table 2.** Transcriptomic Blood Biomarkers

Authors	Initial blood biomarker	Potential blood biomarker
Oh et al.^21^	mRNA from Blood	CN vs. MCI (17 genes), CN vs. AD (10 genes), MCI vs. AD (16 genes)
Yasodya et al.^22^	mRNA from Blood	100 influential AD-associated genes
Xu et al.^16^	miRNA from Serum and Plasma	APP, BACE1, MAP2K1, GSK3B, and AKT3
Yuan et al.^3^	mRNA from Blood	STAT6, KLF6, FCER2, HLA-A, PPBP
Chiricosta et al.^23^	mRNA from Blood	ATF4, ATP5PF, AXIN1, CDK5, COX7A2L, EIF2AK2, ERN1, FZD2, HRAS, LRP6, NDUFB22, NDUFS5, NDUFS7, NFKB1, PSMA5, PSMD1, PSMD8, SDHA, SDHC, SDHD, SLC39A9, WNT4, XBP1
Kelly et al.^24^	mRNA from Blood	159 genes
Huseby et al.^11^	mRNA from Blood	MRPL51, NDUFA1, NDUFS5, RPL36AL, LOC646200
Shvetcov et al.^25^	mRNA from Blood	LSM3, RPS27A
Abdullah et al.^13^	mRNA from Blood	CST7, DPAGT1, DZIP1, MOSC1, TACC3, YDJC
Abdullah et al.^4^	mRNA from Plasma	ANKRD28, CCDC92, DEFA3, FBXO32 GRIA4, HDAC7, IFITM3, LY6G6D, MC1R, RPL18, SPOCD1, ST14, TOR1AIP2, TRIM16L, UBXN7, VEGFB
Zhao et al.^26^	miRNA from Serum	hsa-miR-346, hsa-miR-345-5p, hsa-miR-122-3p, hsa-miR-208b-3p, hsa-miR-499a-5p, hsa-miR-206, hsa-miR-1246, hsa-miR-145-5p, hsa-miR-650, hsa-miR-1285-3p, hsa-miR-208b-3p, hsa-miR-206
Zhang et al.^10^	mRNA from Blood	Chromosomal Regions: Chr.19p12-19p13.3, Chr.1p22.1-1p31.1, Chr.1q21.2-1q23.1. Transcription Factors: E2F2, FOXO3, GATA1. Biological Insights: Dysregulation in immune responses, hematopoiesis, exocytosis, and neuronal functions.
Liu et al.^27^	miRNA from Plasma	nine, two, and eight miRNAs significantly associated with A/T/N
Karpagam et al.^28^	miRNA from Blood	None
Dinuwanthi et al.^2^	miRNA from Blood	hsa-miR-186-5p, hsa-miR-144-3p, hsa-miR-151a-3p, hsa-miR-99b-5p, hsa-miR-98, hsa-miR-148a-3p, hsa-let-7g-5p, hsa-let-7a-5p, hsa-miR-30d-5p, hsa-miR-15a-5p, hsa-miR-589-5p, hsa-miR-144-5p, hsa-let-7f-5p, hsa-miR-4781-3p, brain-miR-112, hsa-let-7a-5, hsa-miR-148b-5p, hsa-miR-29b-3p, brain-miR-431, hsa-miR-378a-5p, hsa-miR-548h-5p, hsa-miR-3909, hsa-miR-625-5p, hsa-miR-24-3p

**Table 3.** Machine Learning Approaches for Transcriptomic Biomarker Analysis

Dataset	Samples	Features	Feature selection	ML models	Best model	Performance metrics
NNI^21^	254	176	Boruta	RF	RF	Accuracy:0.596
ADNI^22^	N/A	11,276	VAE	RF, SVM, LR, DNN, L1-LR, VAE	VAE-LR	AUC:0.760
Curated miRNA set^16^	188	22,984	InfoGain	MLP, NB, RT, RF, ZR	N/A	Serum accuracy: 0.920,Plasma accuracy: 0.909
GEO^3^	711	5,625	RFECV	SVM, Adaboost, RF and ANN	SVM	Accuracy:0.916, AUC:0.975
GEO^23^	180	23,716	Univariate selection	LR, DT, Gaussian NB, k-NN, RF, ANN, SVM	SVM	Accuracy: 0.890, AUC: 0.930
GEO^24^	515	19,147	VSSRFE	LR, SVM, XGBoost, RF, MLP, VAE	RF	Accuracy:0.810, AUC:0.889
GEO^11^	3,490	23,240	RF	RF	RF	AUC:0.800
GEO^25^	736	N/A	RFE	RF	RF	Sensitivity:0.670, Precision:0.770
GEO^13^	453	47,008	Boruta	lasso-LR	lasso-LR	Sensitivity:0.631, Specificity:0.631, Accuracy:0.684, Error rate:0.316, AUC:0.763
TUA^4^	184	42,546	Boruta	Lasso LR, Ridge LR, Elastic Net LR, NB, SVM-RBF, k-NN	Elastic net-LR	Accuracy:0.816, Sensitivity:0.852
OPTIMA^26^	96	566	RFE	RF	RF	Accuracy:0.857, Sensitivity:0.889, Specificity:0.800.
ADNI, ANM^10^	1,452	N/A	Differential expression	XGBoost	XGBoost	AUC:0.810, Sensitivity:0.810, Specificity:0.630
ADNI^27^	803	336	DESeq2 LRT	NB, LR, elastic net, DT, RF, GB, SVM, ANN, k-NN	RF	AUC for Amyloid: 0.680,Tau: 0.620,Neurodegeneration: 0.640
GEO^28^	70	506	None	J48 Decision Tree, RF, SVM, LR, k-NN, NB	RF	AUC:0.927
GEO^2^	70	2,652	Statistical Filtering, PCA,RF, and Correlation	RF, LR, SVM	RF	Accuracy:0.952, Sensitivity:0.941, Specificity:0.875, AUC:0.880

Oh et al^21^ employed gene expression data from mRNA for AD classification. The study used a cohort of 254 participants with 176 features from the NNI dataset comprising 99 cognitively normal (CN), 61 MCI, and 94 AD. The authors also performed validation with the external ADNI dataset. The authors focused on a panel of 176 inflammatory genes related to neuroinflammation. The authors applied the Boruta algorithm to find the significant genes. The study identified 17 genes that differentiate CN individuals from those with MCI, 10 genes that distinguish CN from AD, and 16 genes that classify MCI from AD. RF models were used to handle non-linear data. Moreover, the RF model showed accuracy of 0.596 for CN vs MCI, 0.560 for CN vs AD, and 0.567 for MCI vs AD. The selected genes identified signaling pathways such as PI3K-Akt, IL-17, JAK-STAT, TNF, and Ras. Yasodya et al^22^ used mRNA from blood samples to identify optimal features for AD prediction. The authors processed 49,386 probes to obtain 11,276 genes. The authors used TF-related genes, hub genes, CFG scoring, and a Variational Autoencoder (VAE)^29^ to reduce feature dimensionality. The study identified 100 AD-related genes that contributed to improved diagnostic accuracy. The authors used ML techniques such as RF, SVM, Logistic Regression (LR),^30^ Deep Neural Networks (DNN),^31^ and L1-regularized Logistic Regression (L1-LR).^32^ The proposed VAE-LR model showed the highest area under the curve (AUC) value of 0.760. The study proved that 100 identified genes exhibit insights into molecular mechanisms underlying AD pathogenesis. Both studies^21,22^ used blood-based mRNA and RF models to investigate Alzheimer’s disease. However, the study^22^ achieved a higher AUC of 0.760 than the study.^21^

Xu et al^16^ investigated miRNA blood biomarkers from plasma and serum samples to develop an optimal classification model for AD prediction. The authors used 11,731 attributes for serum and 11,253 for plasma. They applied InfoGain attribute filtering with a threshold of 0.0306 for serum and 0.07 for plasma. 704 serum-based and 54 plasma-based miRNA biomarkers were identified that enhanced AD diagnostic performance. The study used Multilayer Perceptron (MLP),^33^ Naive Bayes (NB),^34^ Random Tree (RT), RF, and ZeroR (ZR)^35^ for AD classification. The model achieved an accuracy of 0.920 for serum samples and 0.909 for plasma samples. The study identified significant genes such as APP, BACE1, MAP2K1, GSK3B, and AKT3. Yuan et al^3^ developed diagnostic models using blood mRNA expression data. The study used 711 samples and 5,625 genes from the GEO dataset. 355 optimal mRNA features were identified by applying Recursive Feature Elimination Cross-Validation (RFECV). The authors evaluated SVM, AdaBoost, RF, and artificial neural networks (ANN)^36^ for AD prediction. The SVM model achieved the highest accuracy of 0.916, with AUC values of 0.975, 0.954, and 0.981 for classifying CN, MCI, and AD cases, respectively. The study identified five biomarkers, such as STAT6, KLF6, FCER2, HLA-A, and PPBP. Both studies^3,16^ achieved high predictive performance with an accuracy near 0.920 for AD diagnosis using blood-based transcriptomic biomarkers. However, the study^16^ utilized miRNA, while the study^3^ used mRNA with RFECV feature selection to select five key genes using an SVM classifier.

Chiricosta et al^23^ developed a model using mRNA extracted from peripheral blood cells. The study exhibited the importance of oxidative stress and mitochondrial pathways. The study used 180 samples with 90 AD and 90 control samples. Feature selection was performed using univariate selection with mutual_info_classif and f_classif. Several ML algorithms were evaluated, including LR, linear discriminant analysis (LDA), decision tree (DT),^37^ NB, k-NN,^38^ RF, neural networks, and SVM. The SVM model outperformed the others, achieving an accuracy of 0.890 and an AUC of 0.930. The study identified 23 key genes, such as ATF4, ATP5PF, AXIN1, CDK5, COX7A2L, EIF2AK2, ERN1, FZD2, HRAS, LRP6, NDUFB22, NDUFS5, NDUFS7, NFKB1, PSMA5, PSMD1, PSMD8, SDHA, SDHC, SDHD, SLC39A9, WNT4, and XBP1. These genes play significant roles in oxidative stress and mitochondrial pathways and are involved in mitochondrial translation, mitochondrial gene expression, and the Alzheimer’s disease pathway (hsa05010). Kelly et al^24^ introduced a model that utilized optimal gene features for effective AD classification. The study analyzed 515 samples (280 AD patients and 235 controls) and 19,147 genes. The authors applied various feature selection techniques, including least absolute shrinkage and selection operator (LASSO),^39^ variable selection and shrinkage random forest (VSSRFE), and VAE. They evaluated multiple machine learning algorithms, such as LR, SVM, XGBoost, RF, MLP, and VAE. The feature selection process identified 159 optimal genes. RF achieved the highest performance, with an AUC of 0.889 and an accuracy of 0.810. The knowledge-based features were derived from GWAS studies, differentially expressed genes, network modules, and pathway databases, particularly focusing on KEGG Alzheimer’s disease pathways. Both studies^23,24^ used blood-based mRNA for AD diagnosis. The study^23^ achieved a higher accuracy of 0.890 than the study^24^ by focusing on oxidative stress and mitochondrial function.

Huseby et al^11^ analyzed 717 AD-related mRNA samples from 3,490 neurodegenerative samples to identify a subset of key features. The study used 717 samples for AD-specific analysis. They applied RF feature selection to 23,240 initial features to identify the optimal features. The RF model was used for classification and showed the highest prediction accuracy. AD1 and AD2 had the highest AUC values of 0.800 and 0.720. The authors found that MRPL51, NDUFA1, NDUFS5, RPL36AL, and LOC646200 were important for AD prediction. Further, the authors identified 22 transcripts associated with three functions: inflammation, epigenetics, and stress. Shvetcov et al^25^ employed mRNA transcriptomic microarray data from whole-blood samples. The study used Principal Component Analysis (PCA)^40^ for dimensionality reduction and k-means clustering for gene enrichment analysis that extracted the top 1000 genes. RF was employed for classification, while RFE^41^ was used to identify the most informative genes. The authors identified 8 significant features from each cluster. The RF classifier with PC1 provided a sensitivity of 0.670 and a precision of 0.770. However, the PC2 model was linked to metabolic, showing a sensitivity of 0.700 and a precision of 0.770. The study suggests that genes such as LSM3 (involved in spliceosome assembly) and RPS27A (associated with protein synthesis and mitochondrial function) served as key features for AD prediction. Both studies^11,25^ used blood-based mRNA and an RF model for AD classification. The study^11^ achieved a better predictive performance with an AUC of 0.800 than the study^25^ by applying RF feature selection directly to 23,240 genes.

Abdullah et al^13^ analyzed 47,008 mRNA genes to identify blood-based transcription biomarkers for AD prediction. The study used 453 samples, comprising 204 AD patients and 249 control samples. The authors applied Rosner’s outlier detection, variance-stabilizing transformation, and robust spline normalization, and reduced the dataset to 22,373 genes. Boruta’s algorithm was applied to select the top 21 features. The authors used bootstrap under-sampling to balance the dataset. A Lasso LR model was used for the final prediction. The model achieved a sensitivity of 0.631, specificity of 0.631, accuracy of 0.684, an error rate of 0.316, and an AUC of 0.763. CST7, DPAGT1, DZIP1, MOSC1, TACC3, and YDJC were identified as six downregulated biomarkers on further evaluation. These six biomarkers showed p-values less than 0.05, indicating key blood transcription markers for Alzheimer’s disease prediction. Abdullah et al^4^ analyzed 22,254 mRNA genes derived from 184 plasma samples. The authors used preprocessing techniques on 42,546 genes and finalized the 22,254 genes. The Boruta algorithm was used to reduce the original set of 22,254 genes to 68 relevant features. However, Elastic Net-LR was further applied to 68 features and reduced to 16 significant biomarkers. The authors used several models, including Elastic Net logistic regression (Elastic Net-LR), Lasso LR, Ridge LR, RF, SVM with radial basis function kernel (SVM-RBF), k-NN, and NB. The Elastic Net LR model achieved the highest accuracy of 0.816 and a sensitivity of 0.852. The authors identified 16 significant genes, including ANKRD28, CCDC92, DEFA3, FBXO32, GRIA4, HDAC7, IFITM3, LY6G6D, MC1R, RPL18, SPOCD1, ST14, TOR1AIP2, TRIM16L, UBXN7, and VEGFB. Both studies^4,13^ used blood-based mRNA and the Boruta algorithm for feature selection to identify Alzheimer’s disease biomarkers. The study^4^ used 184 samples with Boruta followed by Elastic Net-LR, achieving a higher accuracy of 0.816 than the study.^13^

Zhao et al^26^ examined 566 miRNA signatures from 96 serum samples. The authors used RFE with RF ranking, which identified twelve miRNAs as optimal features for AD prediction. The RF model without postmortem-confirmed (PM) data achieved an accuracy of 0.760, a sensitivity of 0.900, and a specificity of 0.667. The model trained on PM-confirmed 12 miRNA signatures yielded an accuracy of 0.857, a sensitivity of 0.889, and a specificity of 0.800. The study highlighted that miR-346 is involved in the processing of amyloid precursor protein (APP) and amyloid-beta, while miR-206 exhibited reduced levels in AD patients. The identified 12-miRNA signature showed distinct upregulation patterns and downregulation patterns that detect late-stage AD pathological changes. Zhang et al^10^ used transcriptomic biomarkers to improve AD diagnosis. The study applied standard quality control with FDR < 0.1 and | log_2_FC| > 1 filtering, followed by differential expression analysis on 1,452 samples. This process identified 191 genes as optimal biomarkers. The study utilized XGBoost for model training. The GridSearchCV hyperparameter tuning and 5-fold cross-validation were also used to enhance the effectiveness of the proposed model. The proposed model achieved an AUC of 0.810, a sensitivity of 0.810, and a specificity of 0.630. chromosomal regions show key genomic markers including, Chr.19p12–19p13.3, Chr.1p22.1–1p31.1, and Chr.1q21.2–1q23.1. Additionally, the study revealed dysregulation in immune responses, hematopoiesis, exocytosis, and neuronal functions. Both studies^10,26^ used blood-based transcriptomic biomarkers for Alzheimer’s disease prediction. The study^26^ utilized miRNA with RFE-RF feature selection, achieving 0.857 accuracy. In contrast,^10^ used mRNA with XGBoost, reporting an AUC of 0.810.

Liu et al^27^ studied 336 miRNAs from 803 plasma samples in the blood. The authors applied differential expression analysis with DESeq2 LRT and identified nine miRNAs strongly associated with amyloid biomarkers, two miRNAs related to tau biomarkers, and eight miRNAs linked to neurodegeneration. NB, LR, elastic net, DT, RF, gradient boosting, SVM, ANN, and k-NN were used for classification. Moreover, the RF showed the highest AUC values of 0.680 for amyloid, 0.620 for tau, and 0.640 for neurodegeneration biomarkers. The study showed that hsa-miR-483-3p gene was important for amyloid pathology. Karpagam et al^28^ analyzed miRNA sequences from blood samples. The authors used the GEO dataset containing 70 samples of 48 AD and 22 controls. The study processed 506 miRNAs through normalization and randomization. They applied various ML algorithms, including the J48 decision tree (WEKA’s Java implementation of the C4.5 algorithm), RF, SVM, LR, k-NN, and NB. A 10-fold cross-validation was utilized to ensure the reliability of the proposed study. The RF model provided the highest AUC of 0.927, while k-NN also showed strong performance with an AUC of 0.901. Dinuwanthi et al^2^ used next-generation sequencing data to analyze 2,652 miRNA profiles extracted from whole blood samples. The study used 70 samples, comprising 48 AD and 22 controls. The authors processed the data through quantile normalization and read count filtering (>50) to identify 513 highly abundant miRNAs. Statistical analysis reduced the feature set to 219 statistically significant miRNAs. The authors used a combination of PCA, RF, and the Pearson correlation coefficient to identify 25 miRNAs as optimal biomarkers. RF, LR, and SVM models were applied after selecting the features. The RF model provided an accuracy of 0.952, 0.941 sensitivity, and 0.875 specificity, with an AUC of 0.880, demonstrating strong diagnostic potential for AD. All three studies^2,27,28^ used blood-based miRNA and evaluated RF models for Alzheimer’s disease prediction. The study^28^ achieved the highest AUC of 0.927 using 70 samples, compared to 0.880 in study^2^ and 0.680 in study.^27^

In transcriptomic biomarker studies, RF and SVM were the most used algorithms for AD classification. RF provided good predictive performance across multiple transcriptomic studies due to its scalability to handle high-dimensional gene expression data. RF reduces the risk of overfitting by incorporating ensemble learning, creating multiple decision trees and combining the predictions. SVM also showed better results in several studies because SVM is highly effective when the number of features is greater than the number of samples. SVM uses kernel functions that help capture nonlinear relationships in gene expression profiles.

Transcriptomic biomarkers showed a diverse range of performance metrics with an AUC ranging from 0.630 to 0.950 and accuracy from 0.550 to 0.950. mRNA studies provide extensive biological insights into disease mechanisms, but face challenges in feature dimensionality. The studies showed that miRNA biomarkers exhibited better predictive performance than mRNA in several studies. Boruta, VAE, RFECV, LASSO, and MI feature selection techniques were commonly used in transcriptomics studies. The following pathways were overlapped with brain tissues, including neuroinflammation and immune dysregulation, mitochondrial dysfunction, oxidative stress, spliceosome impairment, and altered protein synthesis. The transcriptomics studies focused on capturing dynamic pathway dysregulation and brain-specific transcript information.

#### 4.2.2. Proteomic Blood Biomarkers for AD Detection

The proteomic biomarkers offer valuable insights into disease progression and molecular differences in patients’ blood. Several studies have examined blood protein levels to identify key biomarkers associated with Alzheimer’s disease. In this study, omic discovery and targeted immunoassay protein studies were categorized and discussed. The omic discovery proteomic studies analyze large protein panels to find the optimal protein features. Whereas immunoassay protein studies measure the specific associated proteins. A summary of the proteomic blood biomarkers explored across studies is summarized in Table 4. Several studies used ML algorithms to analyze these protein biomarkers. ML methods enable accurate and efficient AD classification by identifying complex patterns among protein biomarkers and quantifying their concentrations. A summary of machine learning applications in proteomic studies is summarized in Table 5.

**Table 4.** Proteomic Blood Biomarkers for AD Detection

Authors	Initial blood biomarker	Potential blood biomarker
Qin et al^12^	Plasma Protein	A2M, ApoE, BNP, Eot3, RAGE and SGOT, PAPPA, ApoH, C3, IGFP, IL-3, PPP, Cortisol
Zhang et al^14^	Serum and plasma	Serum_IL6,Serum_IL7,Serum_sTNFR1,and Serum_sVCAM1,Plasma_TPO,Plasma_Eotaxin3, Plasma_SAA, Plasma_IL6, Plasma_sTNFR1, Plasma_sICAM1
Abate et al^42^	plasma	U-p532D3A8+
Eke et al^8^	plasma proteins	A2M, ApoE, BNP, Eot3, RAGE, SGOT
Khalil et al^6^	plasma protein	A*β*42/A*β*40 with N-14 and N-15 isotopes
Elsersy et al^1^	plasma protein	None
Prabhakar et al^5^	plasma protein	A2Macro,MDC,IL-18,CD5L,FRTN
Eke et al^43^	plasma protein	A2M, APOE, BNP, EOT3, RAGE and SGOT
Al-Kabi et al^44^	plasma protein	BTC, Calcitonin, Eot3, HBEGF, PAPP-A
Nallapu et al^45^	Blood plasma protein	Plasma p-tau181, Plasma NfL
Zhang et al^46^	Serum protein	IL18, sTNFR1, FABP, I309, TPO, IL5, IL6, IL7, TNFA, A2M, B2M, TNC, SAA, sICAM1, sVCAM1, Factor7
Kim et al^9^	Blood plasma protein	A*β*42
Rasheed et al^47^	plasma protein	None
Zhang et al^7^	plasma protein	Human immunodeficiency virus 1 infection, p53 signaling pathway, phosphoinositide-3-kinase–protein kinase B/Akt signaling pathway.

**Table 5.** Machine Learning Approaches for Proteomics Biomarker Analysis

Dataset	Samples	Features	Features selection	ML models	Best model	Performance metrics
ADNI^12^	359	146	CFS, brute-force	CatBoost, RF, LR, NB, SVM	CatBoost	AUC: 0.930 for CN vs AD, 0.900 for CN vs MCI, and 0.910 for MCI vs AD
TARCC^14^	144	42	RFE	SVM	SVM	Precision:0.833, Accuracy:0.893, Specificity:0.769, AUC:0.960
InveCe.Ab^42^	375	6	None	RT, LR	RT	Specificity:0.941,Sensitivity:0.889
ADNI^8^	356	146	CFS, brute-force	SVM	SVM	Sensitivity:0.920, Specificity:0.810, AUC:0.900
ADNI^6^	622	73	correlation	k-NN, SVM, DT,LR, MLP	MLP	Accuracy:0.890,Sensitivity:0.870
ADNI^1^	622	73	None	DT, NB, GB, k-NN, RF, SVM, FL	FL	Accuracy:0.860, Recall:0.970, Precision:0.780,F1-Score:0.860, AUC:0.950
ADNI^5^	396	146	CFS, Brute-force	SVM	SVM	Accuracy:0.800.
ADNI^43^	166	146	Literature and Statistical filter	SVM	SVM	Sensitivity:0.865, Specificity:0.821, Accuracy:0.850, AUC:0.890
ADNI^44^	422	5 (+age, MMSE)	Univariate, SelectFromModel	GB, AdaBoost, RF, DT, LR, XGBoost, Bagging, k-NN, SVM, NB.	XGBoost	Sensitivity:0.940,Specificity:0.980, AUC:0.990,Accuracy:0.940
ADNI^45^	2013	13	Permutation, Forward Selection	RF	RF	AUC: 0.750
TARCC^46^	300	21	SVM-RFE-LOO	SVM, SVM-RFE, SVM-RFE-LOO	SVM-RFE-LOO	AUC:0.980,Sensitivity:0.940, Specificity:0.933
CNUH^9^	40	1348	Deep Learning	ANN	ANN	Accuracy:0.875
ADNI^47^	1063	133	Dependency dependent, Euclidean	SVM	SVM	Sensitivity:1.000, Accuracy:0.995
EMIF-AD MBD^7^	881	3,635	Univariate LR	Neural Network	Neural Network	AUC: 0.748 for A*β*, 0.662 for p-tau, and 0.710 for t-tau

##### 4.2.2.1. Targeted Immunoassay Protein Biomarker Studies

Khalil et al^6^ employed 622 plasma protein samples from the ADNI dataset. From an initial 73 selected features, further analysis identified four key ones: the N-14 and N-15 isotope peak areas for both A*β*42/A*β*40. The study developed Federated Learning (FL) with MLP for AD classification. The proposed FL-MLP model achieved an accuracy of 0.890 and a sensitivity of 0.870. The approach is biologically grounded in the significance of A*β*42/A*β*40 peptides in AD pathology, using their mass spectrometry peak areas as the primary biomarker input for early detection. Kim et al^9^ proposed a framework for detecting blood proteins using a Surface Enhanced Raman Spectroscopy (SERS) platform. The authors developed a biosensing system capable of estimating protein concentrations with 40 human plasma samples for AD detection. The authors developed a feed-forward neural network with three hidden layers containing 2,500, 1,000, and 100 neurons, respectively. The model achieved an accuracy of 0.875 in classifying HC versus AD patients. This study integrates biosensing techniques with deep learning models, enabling accurate AD prediction. Zhang et al^14^ evaluated the effectiveness of combining plasma and serum biomarkers for AD prediction. The study utilized 300 serum samples and 144 plasma samples from the TARCC cohort. The authors preferred to use a combined dataset of 144 samples with 79 AD and 65 control samples. The authors applied RFE on the 21 proteins from serum and plasma to eliminate redundant features. An SVM model utilized the selected biomarkers to improve prediction accuracy. The study identified four proteins from serum: Serum_IL6, Serum_IL7, Serum_sTNFR1, and Serum_sVCAM1 and six plasma biomarkers: Plasma_TPO, Plasma_Eotaxin3, Plasma_SAA, Plasma_IL6, Plasma_sTNFR1, and Plasma_sICAM1. All three studies^6,9,14^ used blood-based protein biomarkers for Alzheimer’s disease prediction. The study^6^ used plasma samples with Federated Learning-MLP and achieved a higher accuracy of 0.890 than the studies.^9,14^

Abate et al^42^ examined a novel blood biomarker known as U-p532D3A8+ using 375 plasma samples. The authors employed an RT approach to develop a predictive model for AD classification. They used a 10-fold cross-validation and rolling-window procedure to monitor the longitudinal progression of AD. The RF model effectively distinguished between stable MCI and MCI-to-AD progression, achieving a specificity of 0.941 and a sensitivity of 0.889. The study found that misfolding of this biomarker is associated with oxidative stress, inflammation, and amyloid-beta progression. Nallapu et al^45^ proposed a model to predict Alzheimer’s disease progression using 2013 plasma protein biomarkers. The authors analyzed protein sequence patterns and identified plasma p-tau 181 and plasma neurofilament light (NFL) as significant contributors to AD prediction. The study utilized training data from 2013 samples. The authors used Permutation Importance with forward selection for feature selection. The authors employed an RF model trained on blood plasma proteins for AD prediction. The RF model effectively differentiated between stages of AD, achieving a mean AUC of 0.750 for distinguishing CN from AD cases and an AUC of 0.640 for differentiating MCI from AD. Zhang et al^46^ developed a model using serum samples for AD detection. Zhang used 300 samples, which consisted of 150 AD patients and 150 normal controls. The authors identified 16 serum proteins by applying the SVM-RFE-LOO approach on 21 proteins for feature selection. The authors identified biomarkers: IL-18, sTNFR1, FABP, I309, TPO, IL-5, IL-6, IL-7, TNF-*α*, A2M, B2M, TNC, SAA, sICAM1, sVCAM1, and Factor 7. The study provided an AUC of 0.980, sensitivity of 0.940, specificity of 0.933. All three studies^42,45,46^ used blood-based protein biomarkers and RF models for Alzheimer’s disease prediction. The study^46^ achieved the highest performance with an AUC of 0.980 than^45^ using SVM-RFE-LOO feature selection.

##### 4.2.2.2. Omic Proteomic Studies

Qin et al^12^ processed 146 plasma proteins to identify optimal features for AD prediction. In this study, 359 samples were used, comprising 112 controls, 135 MCI patients, and 112 AD patients. The authors applied Correlation-based Feature Selection (CFS)^48^ to identify optimal biomarkers. However, authors identified thirteen significant protein biomarkers, including A2M, ApoE, BNP, Eot3, RAGE, SGOT, PAPPA, ApoH, C3, IGFP, IL-3, PPP, and cortisol. The authors trained several ML techniques, such as CatBoost, RF, LR, NB, and SVM. CatBoost achieved the highest performance with an AUC of 0.930 for CN vs AD, 0.900 for CN vs MCI, and 0.910 for MCI vs AD. Additionally, deep learning (DL) models, such as Convolutional Neural Networks (1D-CNN),^49^ were also tested on protein biomarkers. However, the DL models provided a lower accuracy of 0.830 compared to the traditional ML methods. The study showed that protein changes revealed progression patterns across the AD continuum. SGOT and ApoE showed decreased levels, while Eot3, BNP, and PPP demonstrated increased concentrations. APOH and C3 exhibited U-shaped patterns. Eke et al^8^ analyzed 146 plasma proteins and 356 samples for AD detection. They used CFS and brute force for feature reduction and subsequently trained an SVM model with 10-fold cross-validation on plasma protein data. The SVM achieved a sensitivity of 0.920, a specificity of 0.810, and an AUC of 0.900. The proposed study identified a six-protein panel: A2M, ApoE, BNP, Eot3, RAGE, and SGOT. These proteins are associated with blood-brain barrier damage, inflammation, and metabolic processes. Elsersy et al^1^ compared traditional ML models with Federated Learning (FL)^50^ to enhance AD classification. The authors used 622 plasma samples with 73 features. The pre-processing pipeline included data cleaning, transformation, reduction, splitting, and augmentation. The authors applied several ML models, DT, NB, Gradient Boosting (GB),^51^ k-NN, RF, SVM, and FL. The proposed FL approach achieved a highest accuracy of 0.860, a recall of 0.970, a precision of 0.780, an F1-score of 0.860, and an AUC of 0.950. All three studies^1,8,12^ used plasma-based protein biomarkers for AD prediction. The study^1^ employed Federated Learning with 622 samples and achieved a higher AUC of 0.950 than Refs. 8 and 12.

Prabhakar et al^5^ investigated blood protein biomarkers for the AD classification. The authors analyzed 396 plasma protein samples. CFS feature selection techniques were employed that reduced the initial 146 features to 18 proteins. Furthermore, the authors applied brute-force and extracted only five optimal features. An SVM model with a 3-degree polynomial kernel using these features, achieving the highest accuracy of 0.800. The Authors identified A2Macro, MDC, IL-18, CD5L, and FRTN as the five most predictive protein biomarkers for AD classification. Eke^43^ developed a predictive model for effective AD classification using 146 plasma proteins. The study utilized 166 participants, comprising 108 AD patients and 58 healthy controls. The study followed a two-phase feature selection approach. In the first stage, 31 proteins were preselected from the literature review. In the second stage, 14 protein biomarkers were extracted by applying a statistical filter with p-value less than 0.05. The proposed SVM model achieved a sensitivity: 0.865, specificity: 0.821, accuracy: 0.850, and an AUC: 0.890. The most significant predictors identified were Alpha-1 microglobulin, Alpha-2 macroglobulin, Complement C3, Immunoglobulin M, and Tenascin C. The study revealed that a selected protein panel is involved in immune and inflammatory pathways. Al-Kabi et al^44^ developed a model to identify an optimal panel of blood biomarkers. The authors used 148 plasma proteins and 422 subjects from the ADNI cohort. The authors employed a univariate filter and model-based feature importance for feature selection. The study used ML techniques such as GB, AdaBoost, RF, Extra Trees, Feature Transformation (FT),^52^ LR, XGBoost, Bagging, k-NN, SVM, and NB. The XGBoost model achieved the best results with an accuracy of 0.940, a sensitivity of 0.940, a specificity of 0.980, and an AUC of 0.990. The study identified five key protein biomarkers: Betacellulin (BTC), Calcitonin, Eotaxin-3 (EOT3), Heparin-Binding EGF-Like Growth Factor (HBEGF), and Pregnancy-Associated Plasma Protein A (PAPP-A). All three studies^5,43,44^ used plasma-based protein biomarkers and SVM models for Alzheimer’s disease classification. The study^44^ achieved the highest accuracy of 0.940 using XGBoost on 422 samples, compared to 0.850 in study^43^ and 0.800 in study.^5^

In reference 47, Rasheed et al examined 190 plasma proteins to enhance AD classification using blood biomarkers. The set of 146 plasma proteins with 1063 samples was obtained after removing incomplete entries from the dataset. Data augmentation techniques were also considered, including oversampling and undersampling,^53^ to balance the target classes. The authors employed feature selection techniques such as Dependency-dependent methods (Mutual Information, Symmetric Uncertainty, and Cramér’s V) and Euclidean distance-based feature selection. The Dependency-dependent SVM model, utilizing a panel of 22 biomarkers, yielded the best prediction performance, achieving an accuracy of 0.806 and a sensitivity of 0.912. In contrast, the Euclidean distance-based method combined with SVM, employing a larger panel of 133 biomarkers, achieved a sensitivity of 1.000 and an accuracy of 0.995. Zhang et al^7^ analyzed 3,635 plasma proteins from 881 participants to identify biomarkers associated with amyloid, tau, and neurodegeneration. Neural networks with three hidden layers (8-16-8 nodes) and dropout were used for AD classification. The authors selected the top 1000 protein features using the LR model. The baseline model achieved AUCs of 0.748 for A*β*, 0.662 for p-tau, and 0.710 for t-tau. The study identified 35 protein biomarkers that are involved in five major AD-related biological pathways, including human immunodeficiency virus 1 (HIV-1) infection, p53 signaling, and the phosphoinositide 3-kinase–protein kinase B (PI3K–Akt) signaling pathway. The studies^7,47^ used plasma-based protein biomarkers for Alzheimer’s disease classification. The study^47^ analyzed 190 proteins and achieved an accuracy of 0.995. In contrast,^7^ analyzed 3,635 proteins from 881 samples and achieved moderate AUC between 0.662 and 0.748.

In proteomic blood biomarkers, the SVM algorithm was frequently used in many studies. The SVM algorithm provides better performance results in proteomic biomarkers because it performs effectively in high-dimensional spaces. The main focus of SVM is to maximize the margin between the classes, that diminish the risk of overfitting. However, tree-based ensemble models such as XGBoost and CatBoost also achieved better performance metrics. The CatBoost algorithm uses good default parameters that help in AD classification without intensive fine-tuning of hyperparameters. Also, CatBoost uses an ordered boosting approach that reduces the overfitting problem. Moreover, XGBoost uses a regularization technique that helps in the prevention of overfitting, which improves AD classification.

The proteomic biomarkers showed the highest predictive performance among all biomarkers, with an AUC ranging from 0.900 to 0.990. The core AD-pathology proteins (p-tau181, NfL, A*β*42/A*β*40) are measured using sensitive immunoassays. The selected targeted panels of 5 to 20 proteins outperformed hundreds of proteins in most studies. In these studies, various feature selection techniques were employed, including RFE and CFS, to extract the optimal protein feature set. However, proteomics studies exhibited stronger robustness to batch effects and platform differences than transcriptomics biomarkers. The performance metrics vary considerably based on feature selection methods and sample size, with larger panels not always yielding better predictions.

#### 4.2.3. Multi-Omics Blood Biomarkers for AD Detection

In a multi-omics approach, multiple blood biomarkers, transcriptomics, proteomics, metabolomics, and clinical variables are integrated to enhance AD prediction. Several biomarkers from blood were examined together to find patterns of AD biology to overcome the limitations of individual biomarkers. Five studies examined gene expression data along with proteomic, clinical, and metabolic biomarkers from blood samples. Detailed descriptions of these multi-omics studies are summarized in Table 6.

**Table 6.** Multi-Omics Blood Biomarkers for AD Detection

Authors	Initial blood biomarker	Potential blood biomarker
karaglani et al^54^	miRNA, mRNA, Proteins	miRNAs (3), mRNAs (6),Proteins (7)
Jia et al^55^	Plasma Protein,Plasma metabolites	P-tau protein and plasma NfL
Ngai et al^56^	Genomic,Metabolomic, Proteomic	APOE*ɛ*4, GFAP, CXCL17
AlMansoori et al^57^	Gene expression,SNP data	axon myelination and synaptic vesicle membrane
Khanal et al^15^	Gene expression data and SNP data	Gene: XRCC5, RAB27A, PIP4K2A, PSMF1, CCDC176, BNIP3L, OSBP2, TTBK2, IRAK3 SNP-related Genes: TOMM40, RIT2, PDZD2, NPAS3

Karaglani et al^54^ examined proteomic and gene expression data from blood samples to enhance AD prediction. Separate datasets were used for each biosignature from blood. The study used a proteomic dataset with 62 subjects, an mRNA dataset with 234 subjects, and a miRNA dataset with 70 subjects. The proposed model was fully automated using the JADBIO platform. The authors applied ML models, including SVM, RF, and Ridge Logistic Regression. The SVM model achieved the highest AUC values of 0.975 for the miRNA dataset, the Ridge-LR model achieved an AUC of 0.921 for the proteomic dataset, and the RF model achieved an AUC of 0.846 for the mRNA dataset. The multi-omics analysis is illustrated in Table 7. The study selected seven biomarkers from the proteomic dataset, including LRRIQ2, CAMLG, IL4, TPM1, IL20, DIABLO, and VRK3. Additionally, six significant biomarkers were identified from the mRNA data. However, three miRNA biomarkers, MIR29C, MIR30D, and MIR182, were relevant for AD prediction. Jia et al^55^ developed a model for AD classification by combining plasma protein and metabolomics data. The study analyzed three datasets and incorporated sociodemographic and health data from 482 samples. The authors learned temporal relationships by using LR, SVM, DT, RF, XGBoost,^58^ and ANN models. The SHAP explainable model identified five predictors as blood p-tau, plasma NfL, age, blood total tau, and education level. LR effectively learn the complex patterns with an AUC value of 0.818 and a sensitivity of 0.801. The authors selected phosphorylated tau (p-tau), plasma neurofilament light chain (NfL), and total tau as key biomarkers from the plasma proteins. The metabolomics analysis identified taurine, inosine, xanthine, and L-glutamine. The findings suggest that p-tau and plasma NfL are highly reliable biomarkers for the early identification of AD. Both studies^54,55^ used multi-omics approaches for Alzheimer’s disease prediction. The study^54^ achieved high AUCs across all omics types (0.975 for miRNA, 0.921 for proteomic, 0.846 for mRNA) compared to the study,^55^ which achieved an AUC of 0.818.

**Table 7.** Machine Learning Approaches for Multi-Omics Biomarker Analysis

Dataset	Samples	Features	Features selection	ML models	Best model	Performance metrics
BioDataome, Metabolomics Workbench, GEO^54^	Proteomic:62; mRNA:234, miRNA:70	Proteomic:9,483, mRNA:38,327; miRNA:506	JADBIO	SVM,RF, Ridge-LR	N/A	AUC for miRNA:0.975, AUC for proteomic:0.921, AUC for mRNA:0.846
ADNI^55^	1,219, 863, 482	11, 17, 23	None	LR,SVM, DT,RF, XGBoost, ANN	LR	AUC:0.818,Sensitivity:0.801
UK BioBank^56^	3,727	5,553	None	LGBM, CatBoost, XGBoost, TabNet,and LR	CatBoost	AUC: 0.870
ADNI^57^	623	Gene:19,403, SNP:2.7 million	Chi-square, MI, LASSO	AdaBoost, MLP,RF, SVM	RF	AUC:0.940,Accuracy:0.950
ADNI^15^	Gene:743, SNP:519	Gene:49,386, SNP:294,221	Statistical filters, model-based wrapper	RF,GB, XGBoost	XGBoost	AUC with gene:0.650, AUC with SNP:0.640

Ngai et al^56^ examined blood-derived modalities to determine AD blood biomarkers. The authors analyzed 3,727 subjects and 5,553 features in intersection datasets based on genomic, proteomic, metabolomic, and medication data. They used various ML techniques, including Light Gradient Boosting Machine (LGBM),^59^ CatBoost, XGBoost, TabNet, and LR. Among these, the CatBoost model achieved the highest AUC value of 0.870 for AD prediction. Their analysis showed that genomic and proteomic biomarkers provided informative insights compared to the other modalities. They identified APOE*ɛ*4, GFAP, and CXCL17 as highly predictive biomarkers for AD diagnosis. AlMansoori et al^57^ developed a framework using gene expression, single-nucleotide polymorphism (SNP) data, and clinical features. The study used 2,702,858 features from SNPs and 19,403 genes from the gene expression dataset. The study employed Chi-square, Mutual Information (MI), and LASSO to handle high-dimensional data. The authors used ML pipelines such as AdaBoost, MLP, RF, and SVM for AD classification. The RF model MI technique achieved an AUC of 0.940 and an accuracy of 0.950. The AdaBoost model utilizes feature selection on SNP data, resulting in an AUC of 0.550 and an accuracy of 0.600. The authors demonstrated that genetic biomarkers involved in axon myelination and synaptic vesicle membrane formation offer valuable insights into AD pathology. Khanal et al^15^ analyzed gene expression and SNP data from blood samples to enhance the AD prediction. In this study, 743 subjects for gene expression and 519 for SNP analysis were extracted from the ADNI cohort. The statistical filters and model-based wrapper feature selection technique were derived to find the most predictive features. SMOTE was incorporated to address the class imbalance in the dataset. In reference,^15^ the authors used RF, Gradient Boosting, and XG-Boost to identify the best-performing model for AD classification. The gene expression with age and education features provided an AUC of 0.650. The study identified gene expression features, XRCC5, RAB27A, PIP4K2A, PSMF1, CCDC176, BNIP3L, OSBP2, TTBK2, and IRAK3. The SNP genes identified were TOMM40, RIT2, PDZD2, and NPAS3. The authors concluded that gene expression profiles provided better accuracy than SNP-based biomarkers. All three studies^15,56,57^ used multi-modal approaches combining multiple blood-based biomarker types for Alzheimer’s disease prediction. The study^57^ analyzed 2,702,858 SNP features and 19,403 genes and achieved a superior AUC of 0.940 and accuracy of 0.950 compared to the study Refs. 15 and 56.

In multi-omics biomarker studies, various ML algorithms achieved superior performance metrics in AD classification. RF performed better in multi-omics because it combined the predictions of multiple decision trees, which reduced the risk of overfitting. RF used an ensemble learning approach that improved the ability to learn a nonlinear decision boundary. Furthermore, SVM provided better performance metrics because SVM performed well in high-dimensional spaces. SVM utilized kernel functions that effectively captured non-linear relationships. The CatBoost algorithm was also effective in multi-omics because it required minimal hyperparameter tuning and used symmetric trees that improved AD classification. However, XGBoost demonstrated weak performance in multi-omics studies because it required careful parameter tuning to achieve good performance. XGBoost was also prone to overfitting if regularization was not handled properly.

The multi-omics studies integrating transcriptomic, proteomic, genomic/SNP, metabolomic, and clinical biomarkers reported AUCs ranging from 0.650 to 0.976. Feature selection techniques including JADBIO AutoML, Chi-square, Mutual Information, and LASSO effectively reduced high-dimensional data to highly predictive features. These studies identified key biomarkers, including APOE*ɛ*4, GFAP, p-tau and NfL. The integration of clinical variables like age and education with molecular data was incorporated in several studies to enhance prediction. However, multi-omics studies face significant challenges, including high feature dimensionality and variable performance across omic layers. These findings highlight the potential of multi-omics blood panels for improving Alzheimer’s disease diagnosis.

#### 4.2.4. Elemental and General Clinical Blood Biomarkers for AD Detection

The elemental and clinical biomarkers linked to the progression of Alzheimer’s disease. These biomarkers show metabolic and inflammatory changes that may help detect neurodegenerative disease. This review examines two studies that investigate these blood biomarkers. Table 8 provides an overview of elemental and general clinical blood biomarkers.

**Table 8.** Elemental and General Clinical Blood Biomarkers for AD Detection

Authors	Initial blood biomarker	Potential blood biomarker
Hasan et al^60^	General Blood Biomarkers	Glucose levels, total protein, and RBC count
Safi et al^61^	Na, K, Mg, Ca, Fe, Zn, Mn, Cu, Cr, and Se	Na/K, Fe/Na and P/Zn

Hasan et al^60^ investigated 57 blood parameters from 1004 subjects for AD prediction. They applied feature selection methods, including Boruta, ANOVA, and Pearson correlation, to identify the 26 features. These significant biomarkers included clinical parameters such as glucose levels, total protein, and red blood cell (RBC) count. The authors used SVM, LR, Gaussian naive Bayes (GNB), and gradient boosting machines (GBM) for the classification of MCI patients using blood samples. Five-fold cross-validation was employed to enhance predictive accuracy. LR showed the highest performance, achieving an accuracy of 0.648 and an AUC value of 0.676. The comprehensive details regarding the usage of ML models on elemental and general clinical blood biomarkers are discussed in Table 9. Safi et al^61^ used elemental biomarkers and used 59 plasma samples to calculate the amount of Na, K, Mg, Ca, Fe, Zn, Mn, Cu, Cr, and Se. The author applied a correlation filter to avoid multicollinearity. The SVM model with a linear kernel and Leave-One-Out Cross-Validation (LOOCV) was used to examine the concentration of elemental biomarkers. However, three elemental ratios, such as Na/K, Fe/Na, and P/Zn, achieved an accuracy: 0.810, specificity: 0.790, and sensitivity: 0.840. Both studies^60,61^ used blood-based biomarkers for Alzheimer’s disease prediction. The study^61^ used elemental biomarkers from only 59 plasma samples and achieved a higher accuracy of 0.810 with SVM and LOOCV than the study.^60^

**Table 9.** Machine Learning Approaches for Elemental and General Clinical Biomarker Analysis

Dataset	Samples	Features	Features selection	ML models	Best model	Performance metrics
MELoR^60^	1,004	57	ANOVA, Boruta, MRMR, Pearson Correlation, Variance	SVM, LR, GNB, and GBM	LR	Accuracy:0.648, AUC:0.676.
Bedford VA Hospital Dementia Care Unit^61^	59	7	Systematic evaluation, Forward selection	SVM, LR	SVM	Specificity:0.790, Sensitivity:0.840, Accuracy:0.810.

In elemental and general clinical biomarker studies, ML models such as LR and SVM were used for effective AD classification. SVM provided better predictive metrics due to the use of a kernel function to handle high-dimensional space, which improves the ability to deal with complex relationships between features.

Two studies focus specifically on elemental and routine clinical biomarkers for AD prediction. The studies showed an average accuracy ranging from 0.650 to 0.810. However, elemental concentration ratios (Na/K, Fe/Na, P/Zn) provide significant informative patterns that effectively classify more than 0.80 in a small cohort.

### 4.3. Challenges in Using Blood Biomarkers and Machine Learning for Alzheimer’s Detection

This study examines the application of machine learning and blood biomarkers for AD prediction. It analyzes various categories of biomarkers, including transcriptomic, proteomic, multi-omic, and elemental biomarkers. ML models identified features that enhance the accuracy of AD detection. However, ML leveraging blood biomarkers presents notable challenges, particularly due to limited sample sizes. Several studies, including,^7,8,14,23,24,42,54,55,61^ emphasized the issue of small sample sizes. Chiricosta et al^23^ used only 90 AD patients and 90 control subjects, which limited the model’s performance. ML models require large datasets for effective training. Similarly,^8^ used only 146 plasma proteins, resulting in lower model accuracy. Safi et al^61^ analyzed 59 plasma samples comprising 34 healthy controls and 25 AD patients. Such limited sample sizes lead to overfitting, where the model struggles to extract complex relationships between features, reducing its predictive performance. Another significant challenge emphasized by many investigators is the issue of imbalanced datasets. The unequal distribution of classes, such as HC, MCI, and AD patients, complicates the training of models. Imbalanced datasets lead to models achieving high precision but low recall, showing biased and inconsistent predictions. For instance, Ngai et al^56^ experience this issue that imbalanced classes caused the model to be inappropriate for accurate prediction. Similarly, Kelly et al^24^ highlighted how this challenge resulted in higher specificity but lower sensitivity, further contributing to biased outcomes. Another critical challenge noted by Huseby et al^11^ is the absence of standardization across datasets and protocols. The heterogeneous datasets derived from various laboratories and devices introduce inconsistencies in sample values. Disparities in data collection and preprocessing methods resulting from different protocols impact the performance of models. Such inconsistencies slow the development of robust and generalizable predictive models.

Another significant challenge in integrating blood biomarkers with ML techniques is data leakage during feature selection. The application of feature selection before splitting the dataset into training and testing sets may often lead to data leakage. Even when k-fold cross-validation is used, leakage can still occur if preprocessing, resampling, or feature selection is performed outside the cross-validation loop rather than within each training fold. The model utilizes testing data during training, which may limit its ability to generalize and affect the predictive performance. Among the 36 reviewed studies, studies such as Refs. 4, 5, 7, 10, 12, 13, 16, 23, 27, 43, 44 and 60 have used feature selection techniques including Boruta, CFS, t-test, DESeq2, InfoGain, on the entire dataset before cross-validation or data splitting. Additionally, several studies, such as Refs. 2, 5-8, 12, 13, 23, 25-28, 43, 44, 47, 55, 56, 60 and 61 have also applied preprocessing steps like z-score normalization or mean imputation to the entire dataset before splitting. Furthermore, various studies have performed feature selection correctly only after splitting or within cross-validation loops, such as Refs. 11, 15, 21, 24 and 57. For some studies, the order of feature selection was not clearly reported, such as Refs. 1, 3, 14, 22, 45, 46 and 54.

External validation is a common challenge in the literature. Researchers faced difficulties due to the scarcity of independent datasets. The lack of familiar platforms for collecting sample values has increased this challenge in ML-based biomarker research. Evaluating a proposed model on an independent data set is crucial to ensure its generalizability and robustness. However, variations in data collection protocols and insufficient clinical information often limit the clinical acceptance of such models.

Analysis of performance metrics strongly affects the interpretation of clinical applicability. This interpretation may depend on the severity of validation techniques, such as internal validation (cross-validation or a single train/test split from the same dataset), independent test sets (a separate split from the same cohort not used for training), and external validation (a completely different cohort or dataset). The validation techniques used in the included studies were also investigated. Among the 36 studies reviewed, 25 studies utilized internal validation, including Refs. 1, 2, 4-9, 12-15, 22, 23, 25, 27, 28, 42, 43, 46, 55-57, 60 and 61. Moreover, only 7 studies used independent test sets, including Refs. 11, 24, 26, 44, 45, 47 and 54. Similarly, only 4 studies utilized external validation, such as Refs. 3, 10, 16 and 21.

Overfitting is another major challenge that adversely impacts model performance. Studies such as Refs. 14 and 42 highlight this issue. Zhang et al^14^ integrated serum and plasma blood biomarkers to assess predictive performance; however, combining multiple biomarker sources caused overfitting due to variations in patterns and protocols across the datasets. Similarly,^42^ reported that their model learned from random noise rather than meaningful patterns, resulting in overfitting. Thus, overfitting remains a concern when applying machine learning models to blood biomarkers for AD prediction. Several researchers emphasize data heterogeneity as another significant challenge. Studies such as^21,47,60^ highlight that heterogeneity arising from population diversity and individual differences increases model complexity. The inherent noise within samples further impacts model performance. Additionally, variations in sample types, assay methods, and study populations make it challenging for models to identify effective biomarkers. Subtle distinctions in blood biomarkers present another critical challenge in their application. Hasan et al^60^ highlighted the difficulty of distinguishing between HC and MCI due to physiological variations that increase model complexity. This challenge may lead to overfitting, as the model struggles to capture meaningful relationships between features and may consequently overlook significant biomarkers. Additionally,^21^ discussed the difficulty of correlating blood biomarkers with brain pathology in the later stages of AD. As neuronal cell death progresses, differences in blood RNA become less noticeable, complicating AD prediction. Addressing these issues is crucial to enhancing the accuracy of AD prediction models.

## 5. Limitations

The main limitation of this review is the small number of papers included for analysis. In AD research, most studies focus on brain imaging approaches, while blood biomarkers represent an emerging, non-invasive method for diagnosing AD. Including additional biofluid biomarkers could expand the pool of relevant studies.

## 6. Future Directions

Blood biomarkers are an emerging approach with promising potential for integration into clinical practice. To effectively use blood biomarkers for AD diagnosis, standardized protocols are required to mitigate challenges such as overfitting. Increasing acceptance in clinical trials is essential, as blood biomarkers offer a relatively cost-effective and non-invasive alternative to other diagnostic techniques such as MRI, CSF analysis, and PET scans. In this study, only one researcher employed explainable AI to address the black box problem associated with ML models. Future work should incorporate more explainability techniques, such as LIME, to improve the accuracy of AD diagnosis. Moreover, validation using large, independent datasets is crucial to ensuring the robustness and generalizability of these models. Furthermore, the integration of advanced biosensing technologies, such as highly sensitive graphene-based field-effect transistors (GFETs) for biomarker detection, will be crucial for generating the precise and accurate data required to train these robust models. Studies have demonstrated that advanced neural network models outperform traditional machine learning algorithms in AD prediction. DL models consist of an input layer, multiple hidden layers, and an output layer. These models use forward and backward propagation to update the weights, improving predictive accuracy. By iteratively adjusting the weights, DL models minimize the loss function, thereby reducing the model’s error rate. Incorporating additional DL techniques could better capture complex patterns through their multiple hidden layers. Although feature selection techniques are widely applied, hyperparameter tuning remains underexplored, often limiting the model’s potential. More emphasis on hyperparameter optimization is needed to develop more efficient and accurate diagnostic models for AD prediction.

## 7. Conclusion

This review aims to evaluate current research on blood biomarkers and their integration with advanced ML models for predicting AD. The authors systematically collected research papers from multiple publication databases and applied rigorous screening procedures to exclude irrelevant studies. This study identified 36 relevant studies, primarily focusing on blood biomarkers and ML techniques for AD prediction. The review highlights the latest advancements and provides insights into AD prediction. A secondary focus of this study is to classify biomarkers into transcriptomic, proteomic, multi-omics, elemental, and general clinical. Proteomic biomarkers have shown great indicators, including plasma phosphorylated tau 181 (p-tau181), neurofilament light chain (NfL), and glial fibrillary acidic protein (GFAP), achieving AUC values between 0.900 and 0.990. The proteomics biomarkers provide better disease understanding than transcriptomics due to greater stability against batch effects and stronger alignment with AD pathologies, such as tau phosphorylation, neuroaxonal injury, and glial activation. Multi-omics integration further improved the diagnostic performance by capturing temporal patterns from proteomic, transcriptomic, and clinical features. This review indicates the potential of blood biomarkers to shift diagnostic practices from expensive neuroimaging techniques towards less invasive, cost-effective screening in the healthcare sector. This shift facilitates earlier intervention to help delay disease progression. Moreover, the challenges researchers experienced when applying ML algorithms to blood biomarkers include data quality, model validation, overfitting, and biological complexity. The diverse cohorts, independent validation, and explainable AI will be necessary to address these gaps. The integration of blood biomarkers and ML frameworks enhances precision medicine for AD, focusing on reducing diagnostic delays and healthcare burden, and improving early AD detection.

## Data Availability

Data sharing is not applicable to this article as no new datasets were generated or analyzed during the current study.*

## Appendix

### A. Sample Search Strategy

**Table 10.** Database-Specific Search Strategies

Database	Search query
PubMed	(”Alzheimer’s disease”[Title/Abstract]) AND (”blood biomarkers”[Title/Abstract] OR ”blood-based biomarkers”[Title/Abstract]) AND (”Machine Learning”[Title/Abstract] OR ”Artificial Intelligence”[Title/Abstract])
Scopus	TITLE-ABS-KEY(”Alzheimer’s disease”) AND TITLE-ABS-KEY(”blood biomarkers” OR ”blood-based biomarkers”) AND TITLE-ABS-KEY(”Machine Learning” OR ”Artificial Intelligence”)
Web of Science	TS=(”Alzheimer’s disease”) AND TS=(”blood biomarkers” OR ”blood-based biomarkers”) AND TS=(”Machine Learning” OR ”Artificial Intelligence”)
ScienceDirect	TITLE-ABSTR-KEY(”Alzheimer’s disease”) AND TITLE-ABSTR-KEY(”blood biomarkers” OR ”blood-based biomarkers”) AND TITLE-ABSTR-KEY(”Machine Learning” OR ”Artificial Intelligence”)
Google Scholar	”Alzheimer’s disease” AND (”blood biomarkers” OR ”blood-based biomarkers”) AND (”Machine Learning” OR ”Artificial Intelligence”)
IEEE Xplore	(”Alzheimer’s disease”) AND (”blood biomarkers” OR ”blood-based biomarkers”) AND (”Machine Learning” OR ”Artificial Intelligence”)
ACM Digital Library	Abstract:(”Alzheimer’s disease”) AND Abstract:(”blood biomarkers” OR ”blood-based biomarkers”) AND Abstract:(”Machine Learning” OR ”Artificial Intelligence”)

### B. List of Abbreviations

**Table 11.** List of Abbreviations Used in This Review

Abbreviations	Definition
AI	Artificial Intelligence
ANN	Artificial Neural Networks
CFS	Correlation-based Feature Selection
CNN	Convolutional Neural Networks
DL	Deep Learning
DNN	Deep Neural Networks
DT	Decision Tree
Elastic Net-LR	Elastic Net Logistic Regression
FL	Federated Learning
GB	Gradient Boosting
GNB	Gaussian Naive Bayes
K-NN	K-Nearest Neighbors
L1-LR	L1-Regularized Logistic Regression
LASSO	Least Absolute Shrinkage and Selection Operator
LDA	Linear Discriminant Analysis
LGBM	Light Gradient Boosting Machine
LR	Logistic Regression
ML	Machine Learning
MLP	Multilayer Perceptron
NB	Naive Bayes
PCA	Principal Component Analysis
RF	Random Forest
RFECV	Recursive Feature Elimination Cross-Validation
RT	Random Tree
SVM	Support Vector Machine
